# Diabetes App-Related Text Messages From Health Care Professionals in Conjunction With a New Wireless Glucose Meter With a Color Range Indicator Improves Glycemic Control in Patients With Type 1 and Type 2 Diabetes: Randomized Controlled Trial

**DOI:** 10.2196/diabetes.7454

**Published:** 2017-08-07

**Authors:** Mike Grady, Laurence Barry Katz, Hilary Cameron, Brian Leonard Levy

**Affiliations:** 1 LifeScan Scotland Ltd Inverness United Kingdom; 2 LifeScan Inc West Chester, PA United States

**Keywords:** diabetes app, text message, color range indicator, blood glucose monitor, wireless

## Abstract

**Background:**

Mobile diabetes apps enable health care professionals (HCPs) to monitor patient progress, offer remote consultations, and allow more effective and informed treatment decisions between patients and HCPs. The OneTouch Reveal app aggregates data from a blood glucose meter and provides analytics to help patients and HCPs visualize glycemic trends and patterns, enabling more informed treatment and lifestyle decisions. The app also allows patients and HCPs to keep connected by exchanging text messages (short message service [SMS]) or progress reports via email.

**Objective:**

The primary objective of our study was to assess changes in glycemic control and overall experiences of patients and HCPs using the app in conjunction with the wireless OneTouch Verio Flex blood glucose meter.

**Methods:**

We randomly assigned 137 adults with type 1 (T1DM) or type 2 diabetes mellitus (T2DM) and a glycated hemoglobin (HbA_1c_) level of ≥7.5% and ≤11.0% to use the glucose meter alone or glucose meter plus the app for 24 weeks. The meter + app group were scheduled to receive diabetes-related text messages from their HCP every 2 weeks (total of 12 texts). Clinical measures and self-reported outcomes were assessed during face-to-face clinic visits between the participant and a diabetes nurse at baseline, week 12, and week 24.

**Results:**

In 128 completed participants, HbA_1c_ decreased after 12 and 24 weeks in both the meter-only (n=66) (0.56% and 0.55%, respectively) and meter + app groups (n=62) (0.78% and 0.67%, respectively) compared with baseline (each *P*<.001). The difference in HbA_1c_ reduction between the 2 groups was not statistically significant at 12 or 24 weeks (*P*=.12 and *P*=.45, respectively). However, the decrease in HbA_1c_ was greater in T2DM participants using the meter + app after 12 weeks (1.04%) than in T2DM participants using the meter alone (0.58%; *P*=.09). In addition, decrease in HbA_1c_ in participants using the meter + app who received at least 10 diabetes-related text messages (1.05%) was significantly greater than in meter-only participants (*P*<.01).

**Conclusions:**

Use of the OneTouch Verio Flex glucose meter alone or in combination with the OneTouch Reveal diabetes app was associated with significant improvements in glycemic control after 12 and 24 weeks. Improvements using the app were greatest in participants with T2DM and those participants who received the highest number of HCP text messages. This study suggests that real-time availability of patient data and the ability to send personalized diabetes-related text messages can assist HCPs to improve glycemic control in patients between scheduled visits.

**Trial Registration:**

Clinicaltrials.gov NCT02429024; https://clinicaltrials.gov/ct2/show/NCT02429024 (Archived by WebCite at http://www.webcitation.org/6sCTDRa1l)

## Introduction

The advent of mobile phones and smartphones provides a real opportunity to improve diabetes care by enabling patients and health care professionals (HCPs) to exchange information remotely (via text or email) with the potential to minimize or even eliminate the need for routine management office visits [1]. Systematic reviews have found that mHealth interventions improve diabetes care end points such as glycated hemoglobin (HbA_1c_) and are particularly effective if such interventions connect patients with their HCP [2]. mHealth may also facilitate improved engagement in certain patient subgroups, such as adolescents, since a recent Web-based survey showed the most commonly used technology was text messaging (short message service [SMS]) [3]. Although mobile technologies have broad appeal, there is evidence that people with type 2 diabetes mellitus (T2DM) may derive as much, if not more, benefit as people with type 1 diabetes mellitus (T1DM). A meta-analysis showed that mobile phone interventions reduced HbA_1c_ by 0.5% over 6 months, with greater reductions in HbA_1c_ in people with T2DM (0.8%) than in those with T1DM (0.3%) [4]. Furthermore, a review of 13 trials found improved health outcomes in people with T2DM using automated brief messages compared with usual care [5].

Exchanging mobile phone texts or SMS between patients and their HCPs may have an impact on the clinical outcomes of patients. A single-arm study evaluating the effect of SMS text messages on glycemic control in Saudi patients with T2DM found that 5 to 7 texts per week were associated with reductions in HbA_1c_ after 4 months [6]. A study evaluating the effectiveness of daily SMS text messages from a nurse compared with weekly (then biweekly) telephone follow-ups found similar improvements in HbA_1c_ in each group, suggesting that SMS can be considered a valuable method to facilitate diabetes control [7]. Mobile solutions that allow HCPs to remotely visualize patient progress in real time enable HCPs to create personalized SMS text messages containing specific actionable advice. HbA_1c_ was reduced in a study in adults with poorly controlled T1DM or T2DM receiving an average of 13 personalized SMS text messages per week over 3 months [8].

Recent advances in cloud-based diabetes management software and apps have enabled new models of collaborative care between patients and HCPs [9]. We previously reported that using a Web-based version of the OneTouch Reveal app in patients with T1DM and T2DM was associated with a 0.4% reduction in HbA_1c_ after 12 weeks [10]. Certain patients may face other barriers to self-management such as numeracy challenges. Cavanaugh et al [11] described how low diabetes-related numeracy skills are associated with fewer self-management behaviors, and poor numeracy has also been associated with suboptimal glycemic outcomes in both people with T2DM [12] and those with T1DM [13]. The simple color-coded tools used within the OneTouch Verio Flex meter and the OneTouch Reveal app may be especially helpful for these patients. The app contains features such as an easy to personalize reminder to perform self-management activities (eg, medication, physical activity, insulin); graphics showing glucose testing metrics; color coding of low, in-range, or high results (ColorSure Technology); and high- and low-glucose pattern detection tools. The app can also create a 14-day summary report that can be emailed to the HCPs or accessed online by HCPs [14-16].

The primary end point of this study was to evaluate whether use of the app and receiving diabetes-related text messages every 2 weeks from an HCP based on app insights would improve glycemic control in participants with T1DM or T2DM over the 24-week study period. Secondary end points were evaluating text metrics and gathering participant responses to surveys pertaining to acceptance of the meter and the app.

## Methods

### Materials

Participants used a OneTouch Verio Flex blood glucose meter (LifeScan, Wayne, PA, USA); the OneTouch Reveal mobile diabetes app (LifeScan); and a Motorola Moto E smartphone (Basingstoke, UK) preloaded with the app to receive text messages.

### Methods

This parallel 2-arm, open-label, randomized controlled study was conducted at 5 sites in the United Kingdom: Highland Diabetes Institute (Inverness); Edinburgh Royal Infirmary; Queen Elizabeth University Hospital (Glasgow); Heartlands Hospital (Birmingham); and BioKinetics Europe (Belfast). We obtained appropriate ethics approval and participant informed consent before study initiation and registered the trial (NCT02429024; Multimedia Appendix 1 [17]). Participants were existing patients at each clinical site and were identified from the clinic patient databases. Participants were between 16 and 70 years of age; had a diagnosis of T1DM or T2DM for ≥3 months; had a current HbA_1c_ of ≥7.5% and ≤11.0%; and were currently performing self-monitoring of blood glucose (SMBG). All participants received appropriate compensation for time and travel to the clinic site. The primary end point of the study was to determine the HbA_1c_ change from baseline in participants using the meter in conjunction with the app (meter + app) compared with meter-only participants after 12 and 24 weeks. Secondary end points were subgroup analysis of T1DM and T2DM and HbA_1c_ change from baseline at 12 weeks and 24 weeks. Further exploratory end points were the number of texts sent and their association with change in HbA_1c_, and the HCPs’ time to create text messages over 24 weeks. We also explored participant responses to acceptance surveys regarding the meter and app.

#### Visit 1 (Screening)

The first visit was performed 1 week before baseline and included obtaining informed consent, collecting demographic and medical history information, and evaluating inclusion and exclusion criteria. Venous blood was drawn to establish the baseline HbA_1c_ value.

#### Visit 2 (Baseline)

We randomly assigned eligible participants to either the meter-alone or meter + app group. The responsible HCP at each site (diabetes nurse or physician) personalized the color range indicator on the meter for all participants with appropriate low- and high-glucose range limits and gave a full explanation of the meter. Minimum SMBG requirements were recommended based on current therapy (≥1/day for T2DM taking antihyperglycemic agents only; ≥2/day for T2DM on basal or premixed insulin; and ≥3/day for T1DM or T2DM on premixed insulin or multiple daily injections). Participants currently performing SMBG more frequently were encouraged to continue their regimen. HCPs explained all features of the app and ensured it was programmed with color range indicator settings identical to the meter.

#### Home Activities

Participants in the meter-only group were asked to perform SMBG, reflect upon insights provided by the meter, and make any diabetes-related adjustments consistent with their HCP’s advice. Participants in the meter + app group were asked to perform SMBG, reflect upon insights provided by the meter, and frequently (at least weekly) review aggregated SMBG trends, patterns, and insights on the app. These participants also received text messages every 2 weeks from the site HCP containing specific diabetes-related advice or suggested adjustments.

#### HCP Text Messages

**Figure 1:**
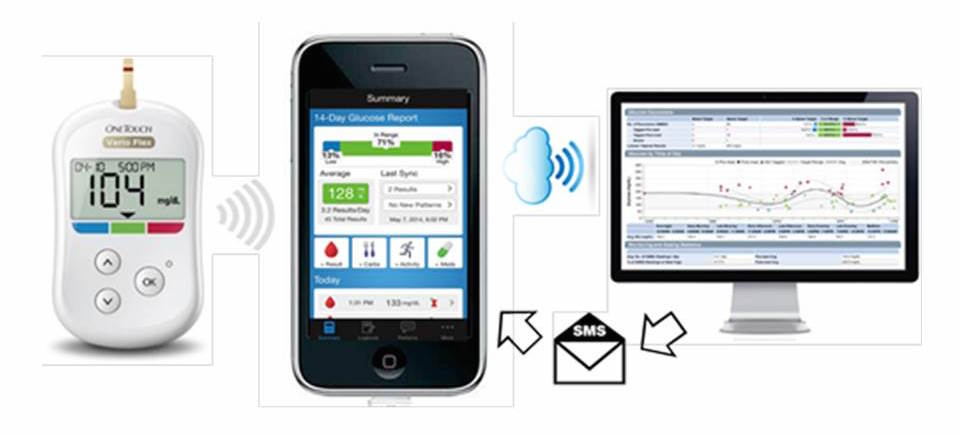
Study data flow. All participants used the OneTouch Verio Flex meter to conduct self-monitoring of blood glucose (SMBG). SMBG data were transmitted wirelessly from the meter to the smartphone containing the diabetes management app OneTouch Reveal. SMBG data were automatically uploaded via the cloud to a Web-based version of the app accessible by site health care professionals (HCPs) on their office computers. HCPs reviewed the 14-day app report for each participant to assist in formulating diabetes-related text messages sent to the participant’s smartphone.

Real-time app data were automatically uploaded (via the cloud) from the participants’ smartphone to a website version of the app accessible by site HCPs on their office computer. A text messaging program (Textlocal; Txtlocal Ltd, Chester, UK) was installed on each HCP’s computer to enable them to easily manage, create, and send texts across multiple participants. HCPs reviewed the 14-day app progress report to assist in formulating diabetes-related text messages sent to the participants’ phone (Figure 1). HCPs completed a log summarizing the content and time taken to create each text message.

#### Visit 3 (12 Weeks)

Venous blood was drawn for HbA_1c_ measurement. HCPs discussed progress with all participants; downloaded from the meter the first 12 weeks of SMBG data (via cable); and collected any adverse events.

#### Visit 4 (24 Weeks)

Venous blood was drawn for HbA_1c_ measurement and the site HCPs discussed progress with all participants. Participants completed surveys regarding their impressions of the meter and app. HCPs downloaded from the meter the last 12 weeks of SMBG data and collected any adverse events.

### Randomization and Statistical Analyses

We randomly assigned 137 participants to the meter-alone or meter + app group within each study site using a stratified block randomization design with 2 stratification variables, each with 2 levels: baseline HbA_1c_ (7.5% to <9.0% or ≥9.0% to 11.0%) and diabetes type (T1DM or T2DM). Using a pooled HbA_1c_ standard deviation of 1.0% from previous mHealth studies (data on file), we estimated sample size at 64 participants per group to achieve 80% power at 5% significance to detect a 0.5% decrease in HbA_1c_. We described continuous demographic variables by median and range (minimum to maximum) or mean and standard deviation. Analysis of covariance was used to assess the mean changes in HbA_1c_ from baseline. Correlations with HbA_1c_ were assessed using the Pearson correlation coefficient and deemed significant at a 5% significance level. We used Minitab v17.0 (Minitab Inc) and IBM SPSS v21.0 (IBM Corporation) for all analyses. We assessed associations between change in HbA_1c_ based on the receipt of per protocol HCP text messages as an exploratory end point. In addition, we analyzed the number of SMS text messages sent by HCPs, including initial observations regarding the content of individual texts. We also completed a full analysis of the meter and app acceptance surveys.

## Results

### Participants

Table 1 shows baseline characteristics of all 128 participants who completed the study; 9 participants either withdrew or were lost to follow-up during the 24 weeks. Meter-only and meter + app participants had similar baseline characteristics, with a mean HbA_1c_ of 8.9% and mean duration of diabetes of about 17 years. Over 70% of participants (94/128) reported performing SMBG ≥3 times per day. Of all 128 participants, 111 (86.7%) were on some form of insulin therapy. The great majority of participants (117/128, 91.4%) had no diabetes apps on their current phone; 122 of 128 (95.3%) had never used diabetes management software; and only 12 of 128 (9.4%) responded that their HCP had ever downloaded SMBG data during consultations.

### Changes in Glycemic Control (HbA _1c_) in all Participants

**Figure 2:**
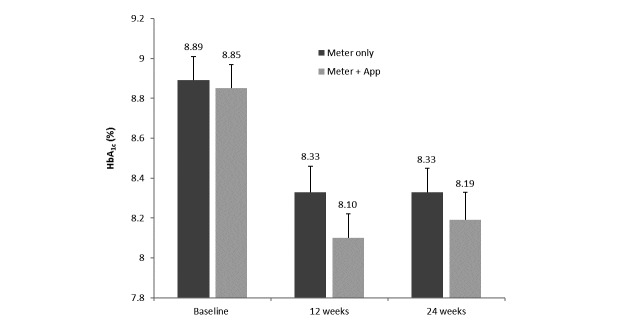
Glycated hemoglobin (HbA_1c_) at baseline and after 12 and 24 weeks of home use for participants using the meter only or the meter + app. Data shown are mean (SEM). Differences from baseline were significant in each group at 12 and 24 weeks (*P*<.001). Differences between the meter-only and meter + app groups were not statistically significant at 12 or 24 weeks.

Figure 2 shows HbA_1c_ at baseline and at 12 and 24 weeks for the meter-only and meter + app groups. HbA_1c_ decreased significantly compared with baseline by 0.56% and 0.55% after 12 and 24 weeks, respectively, in the meter-only group (each *P*<.001). HbA_1c_ was decreased compared with baseline by 0.78% and 0.67% after 12 and 24 weeks, respectively, in the meter + app group (each *P*<.001). Decreases in HbA_1c_ in participants using the meter + app after 12 and 24 weeks were greater by 0.22% and 0.12%, respectively, than in those using the meter alone, but these differences were not statistically significant (*P*=.12 and *P*=.45, respectively).

### Changes in Glycemic Control (HbA _1c_) in Participants With T1DM and T2DM

**Figure 3:**
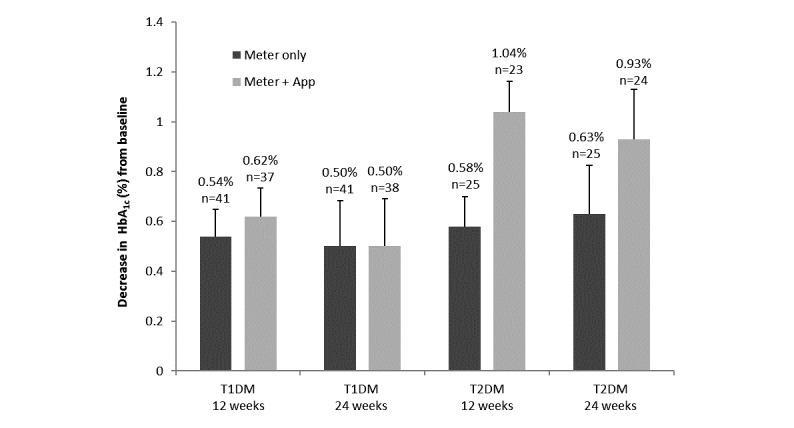
Decrease from baseline in glycated hemoglobin (HbA_1c_) after 12 and 24 weeks of home use in participants with type 1 (T1DM) or type 2 diabetes mellitus (T2DM) in the meter-only and meter + app groups. Data shown are mean (SEM) changes. Differences from baseline were significant in each group at 12 and 24 weeks (*P*<.001). The reduction in HbA_1c_ from baseline was more pronounced in the meter + app group than in the meter-only group, especially at 12 weeks (*P*=.09).

Similar to results in all participants, HbA_1c_ in participants with T1DM decreased compared with baseline both in the meter-only and in the meter + app groups (*P*<.001), and the difference between groups was not significant at 12 or 24 weeks (*P*=.62 and *P*=.98, respectively) (Figure 3). However, in participants with T2DM, the decrease in HbA_1c_ from baseline was more pronounced in participants using the meter + app than in those participants using the meter alone. At 12 weeks, this difference (1.04% vs 0.58%) was significant at *P*=.09 (Figure 3).

### Associations Between Glycemic Control (HbA _1c_) and Text Messaging

**Figure 4:**
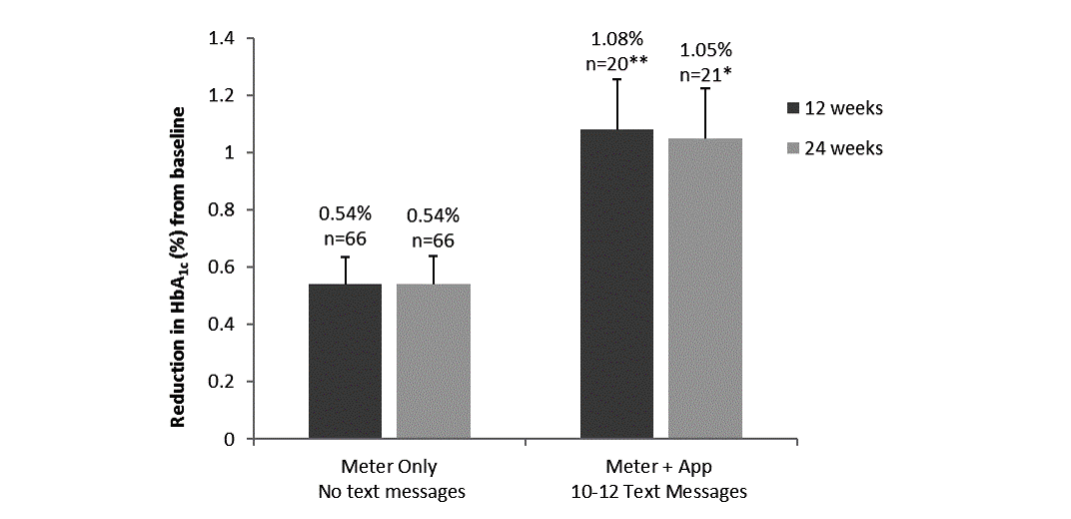
Decrease from baseline in glycated hemoglobin (HbA_1c_) after 12 and 24 weeks of home use for participants who received 10 to a maximum of 12 text messages versus the meter-only group (no text messages). Data shown are mean (SEM) changes. * *P*<.05; ** *P*<.01. Results in the meter + app group were statistically different from the corresponding meter-only group at 12 weeks (*P*<.01) and 24 weeks (*P*<.05).

HbA_1c_ decreased by 1.08% (n=20) in those participants using the app who received at least 10 of the maximum 12 text messages, compared with a 0.54% decrease (n=66) in HbA_1c_ in participants using the meter alone after 12 weeks (Figure 4). This additional HbA_1c_ decrease (*P*<.01) was maintained after 24 weeks. In contrast, there was no difference in the decrease in HbA_1c_ in participants who received fewer than 10 texts compared with participants using the meter alone. Participants (n=21) receiving between 10 and 12 diabetes-related texts from their HCP were sent 223 texts in total over 24 weeks (mean 10.7, SD 0.6 texts) compared with 40 participants receiving 9 or fewer texts (257 texts in total over 24 weeks; mean 6.2, SD 2.4 texts).

**Table 1:** Baseline participant demographics.

Characteristics		Meter only (n=66)	Meter + app (n=62)	All participants (n=128)
**Sex, n (%)**				
	Male	39 (59)	34 (55)	73 (57)
	Female	27 (41)	28 (45)	55 (43)
Age in years, mean (range)		45.1 (20-71)	44.0 (19-69)	44.6 (19-71)
**Diabetes type, n (%)**				
	T1DM^a^	41 (62)	38 (61)	79 (62)
	T2DM^b^	25 (38)	24 (39)	49 (38)
**Hemoglobin A_1c,_mean (range)**				
	All participants	8.9% (7.5%-10.7%)	8.9% (7.5%-10.8%)	8.9% (7.5%-10.8%)
	T1DM	8.9% (7.5%-10.7%)	8.8% (7.5%-10.8%)	8.9% (7.5%-10.8%)
	T2DM	8.9% (7.5%-10.7%)	8.9% (7.5%-10.7%)	8.9% (7.5%-10.7%)
**Duration of diabetes in years, mean (range)**				
	All participants	16.7 (3.9-43.0)	17.1 (3.7-45.4)	16.9 (3.7-45.4)
	T1DM	19.0 (5.1-43.0)	20.5 (3.7-45.4)	19.7 (3.7-45.4)
	T2DM	13.0 (3.9-23.7)	11.8 (4.3-23.0)	12.4 (3.9-23.7)
**Self-monitoring of blood glucose frequency, n (%)**				
	≥5 times/day	12 (18)	13 (21)	25 (20)
	3-4 times/day	39 (59)	30 (48)	69 (54)
	1-2 times/day	13 (20)	13 (21)	26 (20)
	Other	2 (3)	6 (10)	8 (6)
**Treatment therapy^c^for overall/T1DM/T2DM, n**				
	Basal + bolus	46/39/7	38/33/5	84/72/12
	Premix	8/2/6	8/3/5	16/5/11
	Basal only	2/0/2	6/1/5	8/1/7
	Bolus only	1/0/1	2/1/1	3/1/2
	Antihyperglycemic agents only	9/0/9	8/0/8	17/0/17

^a^T1DM: type 1 diabetes mellitus.

^b^T2DM: type 2 diabetes mellitus.

^c^Participants taking insulin may or may not also have been taking antihyperglycemic agents.

### HCP Text Content, and Time and Impact of App-Based Texts on Routine Patient Care

A total of 480 text messages were sent by all HCPs. These texts contained 641 specific instances of advice, including 102 text messages containing advice to adjust bolus or premixed insulin and 84 text messages containing advice on basal insulin. Participants with T1DM and T2DM received similar text advice relating to SMBG. As expected, participants with T2DM received more text advice regarding medications and texts confirming that their diabetes management or SMBG was on track than did participants with T1DM.

The time to review the app 14-day progress report and create a text message was <5 minutes (106/480, 22.1%), 5-10 minutes (244/480, 50.8%), 10-20 minutes (98/480, 20.4%), and >20 minutes (12/480, 2.5%), with 4.2% (20/480) of HCPs not recording times. In terms of managing patients in a clinical setting, the HCPs postulated they would have postponed 12 (9.7%) of the 124 scheduled study visits based on the participant being on track with their diabetes management. Of consultations that would have proceeded, HCPs expected 41 (33%) visits to be shorter and a further 41 (33%) visits to be more focused or to include better-quality conversations given they had the advantage of remote access to glycemic data in advance via the app. If this had been routine clinical practice, in 37 (29.8%) occasions HCPs postulated they would have brought forward this visit earlier or contacted the patient immediately due to concerns identified remotely. Detailed information on the exchange and content of text messages including analytics and experiences of participants using the app will be summarized in a follow-up publication.

### Safety and Tolerability

A total of 60 adverse events and 10 serious adverse events were reported by the 128 participants over the course of the 24-week study. None were related to the meter or the app.

### Participants’ Perceptions of the Glucose Meter and App

Table 2 summarizes participants’ responses to survey statements regarding their opinion of the meter (all participants) and those using the app. Of all 126 participants who responded to the survey, 112 (88.99%) agreed that the simple color range indicator on the meter made it easy for them to manage their blood sugar because they quickly knew whether they were low, in-range, or high; 103 (81.8%) agreed that the low indicator on the meter may help them better manage lows and avoid hypoglycemic events. For app participants, 53 of 56 (95%) agreed that the colorful visuals and pattern messages in the app told them when they were doing well and when they needed to pay more attention; and 53 of 56 (95%) also wished they had had the app when first diagnosed because they felt it would have made their diabetes journey easier. In the 58 meter + app group respondents, 51 (88%) said the simple color range indicator on the meter together with the app could help them to stay on track between visits to their HCP, and 52 (90%) said that the meter and app combination provided a seamless way for them to stay connected with their HCP.

**Table 2:** Participants’ responses to survey statements.

Statement category		n (%)^a^
**OTVF^b^glucose meter (n range 123-126)**		
	The OTVF meter logs my past readings so I don’t have to worry about writing them down	117/124 (94.4)
	The OTVF meter keeps a real-time logbook of my readings that I can carry around with me anywhere	118/125 (94.4)
	It’s reassuring to know that with OTVF I have my blood sugar information at my fingertips	113/125 (90.4)
	OTVF with its simple color range indicator made it easy for me to manage my blood sugar because I quickly knew whether I was low, in-range, or high	112/126 (88.9)
	I think the OTVF meter is for people on the go	109/124 (87.9)
	The OTVF meter makes testing my blood sugar easy so I can get on with my life	107/124 (86.7)
	I found the simple color range indicator feature on OTVF to be a very helpful tool to indicate how I was managing my diabetes	108/126 (85.7)
	I love that the range indicator arrow instantly points to the appropriate color bar after each test so that I quickly know if I am low, in-range, or high	106/125 (84.8)
	The low range indicator on OTVF may help me better manage my lows and avoid hypoglycemic (low blood sugar) events	103/126 (81.7)
	The high range indicator on OTVF may help me better manage my highs and avoid hyperglycemic (high blood sugar) events	100/125 (80.0)
	The color range indicator on the OTVF meter made me feel confident about managing my blood sugar	96/123 (78.0)
	The simple color range indicator on the OTVF meter made it easier for me to follow my HCP’s^d^ recommendations	98/126 (77.8)
	The low indicator on the OTVF meter may help me to avoid hypoglycemic (low blood sugar) events	97/126 (77.0)
**OTR^c^app (n=56)**		
	The colorful visuals and pattern messages in the OTR app tell me when I am doing well and when I need to pay more attention	53 (94.6)
	I wish I had had the OTR app when I was first diagnosed. It would have made my journey easier	53 (94.6)
	OTR app made it easier for me to manage my diabetes than using my meter and a paper logbook	50 (89.3)
	OTR app reduces the tedious work of daily tracking and logging so I can focus on other important things in life	48 (85.7)
	OTR app simplified my daily decisions using my blood sugar information	47 (83.9)
	OTR app is a versatile tool and fits into my lifestyle	47 (83.9)
	OTR app was simple and easy to use	47 (83.9)
	This is a true innovation from OneTouch, a brand that I have come to trust	47 (83.9)
	OTR app helps me get to the big picture fast, right in the palm of my hand	47 (83.9)
**OTVF glucose meter + OTR app (n=58)**		
	The OTVF meter together with OTR app could support me in three ways: in the moment, on the go and over time	53 (91.4)
	The OTVF meter together with the OTR app gave me instant information such as 14 day averages which helped me to discuss my progress with my HCP	52 (89.7)
	The OTVF meter together with the OTR app provided a seamless way for me to stay connected with my HCP	52 (89.7)
	The simple color range indicator on the OTVF meter together with the OTR app could help me stay on track between visits to my HCP	51 (87.9)
	The simple color range indicator on the OTVF meter together with the OTR app could help me be more proactive with my diabetes management	51 (87.9)

^a^Percentages shown are favorable responses defined as a response of “strongly agree” or “agree” on a 5-point scale (5=strongly agree; 4=agree; 3=neither agree nor disagree; 2=disagree; and 1=strongly disagree). All favorable response rates are statistically significant (*P*<.001).

^b^OTVF: OneTouch Verio Flex.

^c^OTR: OneTouch Reveal.

^d^HCP: health care professional.

## Discussion

This study demonstrated improved glycemic control (HbA_1c_) after 12 and 24 weeks in participants using a new blood glucose meter. This reduction in HbA_1c_ with the new meter alone was more than might be reasonably attributed to just being in a clinical study (the Hawthorne effect) and for many patients in our study exceeded the reduction in HbA_1c_ typically observed when switching patients to other new meters. For example, in a study comparing participants with T2DM who were receiving multiple daily injections of insulin either trained on a new meter (Abbott Freestyle Lite) or using flash glucose monitoring (Abbott Libre), HbA_1c_ reductions of 0.31% and 0.29%, respectively, were observed after 24 weeks from a baseline HbA_1c_ of 8.8% [18]. The OneTouch Verio Flex meter used in our study features ColorSure Technology, which has been shown previously to improve the ability of patients to interpret blood glucose readings [15,16], perhaps contributing to the benefits observed in this study. To maximize the benefits of the meter, site HCPs personalized the color feature in terms of low- and high-glucose ranges for that participant and described appropriate actions to consider in response to color information. Participants using the meter may also have derived new insights from color-coded information that translated into therapy or behavioral modifications. It would have been interesting to record the extent of these modifications, perhaps using home diaries, but we did not implement these so as to avoid placing an additional burden on participants. However, in feedback surveys, 78% of participants agreed the meter made it easier to follow their HCP’s recommendations, and over 80% responded that color information on the meter helped them to better manage lows (or highs) and avoid both hypoglycemic and hyperglycemic events.

In the meter + app group, HCPs were able to remotely review participants’ SMBG progress in real time by analyzing app data on their office personal computer. On this basis, they considered how best to respond with appropriate diabetes therapy advice using personalized text messages directly to the participants’ smartphones. The protocol instructed that text messages be sent to participants every 2 weeks to synchronize with the HCP’s review of the 14-day app progress report. However, most participants did not receive the full complement of 12 diabetes-related texts over the 24-week period, although 34% (21/62) did receive 10 to 12 texts. It is possible that this lower than prescribed frequency of text contact may have limited glycemic improvement in the meter + app group compared with the improvements in the meter-alone group. This notion is supported by the decrease in HbA_1c_ observed in app participants receiving at least 10 texts, whereas participants receiving fewer texts did not lower their HbA_1c_ any more than participants using the meter alone.

Text messages based on a review of app data was an important factor in driving improved glycemic control between scheduled consultations by prompting either specific actions (eg, changes to insulin dosing, or suggesting participants reflect on diet, exercise, or SMBG trends) or by suggesting other adjustments. It is worth highlighting that the study population was recruited from hospital-based clinics caring for relatively complex diabetes cases. About 94% (58/62) of app participants were taking some form of insulin, including over 83% (20/24) of participants with T2DM. Therefore, it is not surprising that a high proportion of texts included advice to adjust bolus or basal insulin. There were expected differences between text content for participants with T1DM and T2DM. For example, participants with T2DM received a higher proportion of advice on medications than did participants with T1DM. In clinical practice, a key attribute of HCP text feedback between scheduled visits may be simply to reassure patients and encourage positive patient behaviors that have been observed remotely via real-time access to data. In this regard, it was interesting that the highest proportion of texts to participants with T2DM provided reassurance on progress, explaining that they were essentially on track.

A recent meta-analysis showed that a wide variety of telemedicine solutions (including text messaging) can improve glycemic control and lower HbA_1c_ [19]. Despite evidence of improved glycemic control, there remains concern among HCPs that mHealth connections may contribute an additional burden between scheduled office visits. It was encouraging to discover that in our study over 70% of the HCP app report review and text composition took less than 10 minutes and 22% took less than 5 minutes. Furthermore, the time required to review reports and send texts decreased over time, presumably as HCPs became more proficient using texting software and more adept at reviewing the app summary. We would expect that in routine clinical practice, texting will be patient specific depending on the changing circumstances of each patient, such as transitions to different insulin therapies or adjustments to medications, as well as the patients’ desire for remote contact with their HCP. Allowing HCPs the flexibility to offer a more intensive patient-specific text frequency may further improve clinical outcomes.

Having patients use a mobile app enabled HCPs to visualize real-time participant progress and monitor remotely how well (or otherwise) each participant was doing. With this in mind, we asked site HCPs to assume that each participant was a patient they were managing in routine clinical practice and to consider whether they would have approached their next consultation differently armed in advance with knowledge of the patient’s status. HCP feedback indicated that many visits could have been postponed because the patient was on track. Additionally, one-third said that visits would have been shorter or more focused, with higher-quality conversations during the visit. This feedback highlights the practical value of an mHealth solution, such as our app, and may offset the concern that such solutions increase workload burden. Tools such as this app offer HCPs more flexibility and choice in managing the individual needs of patients with diabetes. Patient engagement with technology will be a key factor to successfully managing diabetes as they consider therapy or behavioral adjustments that could contribute to improvements in glycemic control.

### Study Limitations

It is conceivable that differences in glycemic reductions between the meter + app group and meter-only group were masked by the greater than anticipated decrease in HbA_1c_ observed in the meter-only group. Previous studies have shown the value of color features on the meter [14-16] and, in hindsight, it would have been useful to have an additional group in which participants continued using their current glucose meter. However, given that our participants had significant SMBG experience, we did not anticipate such marked reductions in HbA_1c_ when participants were switched to the new meter. As a further consideration, providing participants with a separate phone to review app insights and receive HCP texts (and to send confirmation or clarifications back to the HCP) may have diminished time spent using the app compared with having the app on the participants’ personal smartphone. However, we sought to ensure a consistent app experience on the phone and minimize any bias resulting from different types of phones. Finally, certain system upgrades occurred during the study period, which resulted in participants having to reload the app and in HCP texts being repurposed to assist participants in ways unrelated to diabetes management. This resulted in a lower number of diabetes-related texts to many participants, which may have compromised their glycemic improvement.

### Conclusion

Using the OneTouch Verio Flex glucose meter alone or in combination with the OneTouch Reveal diabetes app was associated with significant improvements in glycemic control after 12 and 24 weeks. Improvements when using the app were greatest in participants with T2DM and in those who received the highest number of HCP text messages. This study suggests that real-time availability of patient data and the ability to send personalized diabetes-related text messages can assist HCPs to improve glycemic control in patients between scheduled visits.

## Appendix

Multimedia Appendix 1 CONSORT-EHEALTH checklist V1.6.1.

## Abbreviations

HbA _1c_: glycated hemoglobin
HCP: health care professional
SMBG: self-monitoring of blood glucose
SMS: short message service
T1DM: type 1 diabetes mellitus
T2DM: type 2 diabetes mellitus
